# Does a broad‐spectrum cannabidiol supplement improve performance in a 10‐min cycle ergometer performance‐test?

**DOI:** 10.1002/ejsc.12116

**Published:** 2024-05-03

**Authors:** Scott H. Gillham, Lynn Starke, Lauren Welch, Edward Mather, Thomas Whitelegg, Neil Chester, Daniel J. Owens, Theodoros Bampouras, Graeme L. Close

**Affiliations:** ^1^ Research Institute for Sport and Exercise Sciences Liverpool John Moores University Liverpool UK

**Keywords:** anti‐doping, performance, RPE, supplementation

## Abstract

Cannabidiol (CBD) is a non‐intoxicating phytocannabinoid which has been proposed to possess anti‐inflammatory and analgesic properties. Given the potential for perceptions of pain to limit exercise performance, the aim of the present study was to investigate if 3 weeks of daily CBD supplementation (150 mg day^−1^) improved performance in a 10‐min performance‐trial on a cycle ergometer. In a randomized, double‐blind and placebo‐controlled study, 22 healthy participants (*n* = 11 male and *n* = 11 female) completed two 10‐min performance trials on a WattBike cycle ergometer interspersed with a 3‐week supplementation period. Supplementation involved either 150 mg day^−1^ oral CBD or 150 mg day^−1^ of a visually identical placebo (PLA). During trials, ratings of perceived exertion (RPE [6–20]), heart rate (HR) and blood lactate (BLa) were collected every 2 min. Mean power (W) was also taken throughout the exercise at each time point. All data were analyzed using two‐way ANOVAs. There were no significant differences (*P* > 0.05) between CBD or PLA groups for mean power (W) during the 10‐min performance trial. There were also no significant differences (*P* > 0.05) in any of the physiological or perceptual parameters (HR, BLa and RPE) between conditions. Three weeks supplementation of a broad‐spectrum CBD supplement did not improve performance via any change in RPE during a 10‐min time trial on a cycle ergometer, and as such, this evidence does not support the claim that broad‐spectrum CBD supplements could be performance‐enhancing in this exercise modality.

## Highlights

- 3 weeks supplementation of 150 mg/day broad‐spectrum cannabidiol (CBD) does not influence perceived exertion during a 10‐min aerobic performance trial.
- 3 weeks supplementation of 150 mg/day broad CBD does not improve physical performance in a 10‐min aerobic performance trial
- Athletes should continue abstinence of “Off‐the‐shelf” CBD supplements to avoid anti‐doping rule violations from The World Anti‐Doping Agency.

## 1 INTRODUCTION

The cannabis plant contains a diverse profile of chemical compounds including various phytocannabinoids. It has been reported that there are ∼144 constituent phytocannabinoids within this annual herbaceous plant (ElSohly et al., 2017). Of these cannabinoids, the most well‐known are ∆^9^‐tetrahydracannabinol (∆^9^‐THC) and cannabidiol (CBD) with the former best known for its psychotropic and intoxicating effects (Burr et al., 2021). Conversely, CBD has no known intoxicating effects or potential for abuse from a psychoactive standpoint (Iffland & Grotenhermen, 2017). Since the removal of CBD from the World Anti‐Doping Agency (WADA) list of prohibited substances in 2018, the use of cannabis‐related supplements has increased significantly (Docter et al., 2020). For example, it has been reported that 26% of professional rugby players have consumed CBD with 80% of consumers citing enhanced recovery as their primary motive for consumption (Kasper et al., 2020). Additionally, given that there are suggestions that CBD may be anxiolytic (García‐Gutiérrez et al., 2020), it can reduce inflammation (Burstein, 2015), and in some instances reduce sensations of pain (Urits et al., 2020); it is unsurprising that this supplement is becoming increasingly desirable to many athletes.

According to WADA, all cannabinoids (except for CBD) are prohibited in competition. Interestingly, ∆^9^‐THC is reported as an adverse analytical finding (AAF) when levels of its metabolites exceed a urinary threshold of ≥150 ng⋅ml^−1^, whereas in the case of other cannabinoids, no such thresholds exist. Many CBD supplements (particularly broad‐spectrum products) available for purchase off‐the‐shelf (OTS) have been reported to contain quantities of some of these other prohibited cannabinoids posing a significant issue from an anti‐doping perspective (Gurley et al., 2020; Johnson et al., 2022; Liebling et al., 2020). Indeed, it is important to consider the entourage effect of cannabinoids. Specifically, it has been suggested that a wider profile of cannabinoids (termed the “entourage effect”) may be required for a supplement to elicit any of the proposed beneficial effects of cannabinoids (Russo, 2019). To date, there are limited data on the effects of broad‐spectrum CBD products on athletic performance and as such it is difficult to fully establish if these multi‐cannabinoid containing supplements have the potential to enhance performance.

A recent pilot study investigated the effects of an acute dose (300 mg) of synthetic CBD on incremental running to exhaustion, and concluded there to be no changes in perceived exertion (RPE), but some measurable changes in subjective ratings pertaining to participants' pleasure during exercise (Sahinovic et al., 2022). However, no study to date has investigated the effects of CBD on prolonged high‐intensity exercise. Indeed, there are well‐defined links between pain and perceived exertion during exercise, which may partly explain the down‐regulation of pacing schema athletes employ during bouts of intense exercise (Waldron & Highton, 2014). Given that CBD has demonstrated potential to provide analgesia in humans (Argueta et al., 2020) and increase enjoyment during exercise (explained via a proposed analgesic affect) (Sahinovic et al., 2022), CBD may provide an ergogenic effect during exercise which is associated with exercise‐induced pain as seen during high‐intensity exercise. Therefore, the primary aim of the present study was to investigate if 3 weeks of daily CBD supplementation (150 mg⋅day^−1^) had the potential to enhance performance compared to placebo during a 10‐min power test on a cycle ergometer. It was hypothesized that following CBD supplementation, average power would be increased and rating of perceived exertion (RPE) would be reduced compared to placebo.

## 2 METHODS

### 2.1 Participants and study design

**FIGURE 1:**
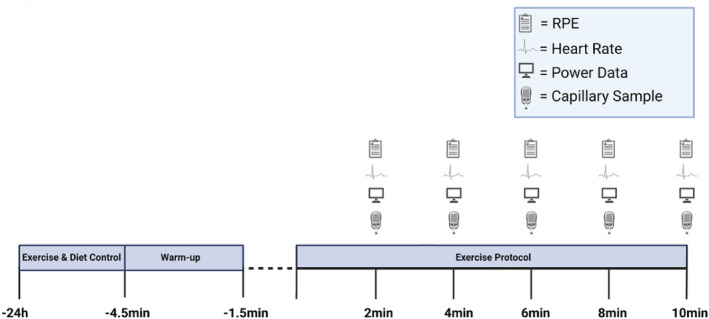
Schematic of experimental procedures.

With institutional Ethics approval (M22_SPS_2273), 24 (male *n* = 12; female *n* = 12) healthy, recreationally active participants (age: 27 ± 6.3 years; stature: 170.4 ± 10.1 cm; body mass: 69.4 ± 13.5 kg) were recruited in a randomized, placebo‐controlled independent groups design. To be eligible for participation in this study, participants had to maintain their normal exercise regime, report no use of prescription medication and were instructed to avoid consuming any cannabis‐related products for 4 weeks prior to and throughout the experimental period. In addition, no participants were involved in any sports that were signatories to the World Anti‐Doping Code at the time of testing. Finally, all participants agreed to using a reliable form of contraception during and 3 months following participation in the study. Upon providing written informed consent, participants completed a health‐screening and readiness to exercise questionnaire and then participants were invited to the laboratory on a minimum of 4 (and maximum of 7) occasions. Visit 1 comprised the collection of anthropometric variables and familiarization with the Wattbike cycle ergometer and exercise protocol. On this initial visit, participants also completed their first complete familiarization 10‐min performance trial. This trial was then repeated on subsequent visits (separated by ∼48 h) until participants were able to achieve a coefficient of variation (CV) < 5% for average power (W), and distance covered (m) during each exercise protocol. When this was achieved, participants were then eligible to begin the experimental period. Participants completed their pre‐supplementation performance‐trial 48 h following their final familiarization visit (Figure 1).

### 2.2 Procedures

#### 2.2.1 10‐min time trial

Participants arrived at the laboratory at the same time of day (±1 h) for each visit to avoid any diurnal variation. All performance trials were completed on a WattBike “Trainer” cycle ergometer (Nottingham, Nottinghamshire, UK). The ergometer flywheel and magnet were set at 5 and 1 for females and 5 and 5 for males, respectively. Before the performance trial, participants were fitted with a Polar H10 heart rate (HR) monitor (Polar Electro, Kempele, Finland) and completed a 3‐min active warm‐up on the ergometer at a self‐elected intensity. Thereafter, participants were provided with a 90s passive recovery before the 10‐min performance trial began. All the exercises were completed with no verbal encouragement or visual feedback from the ergometer. Every 2 minutes, power (W) and distance covered (m) were taken directly from the ergometer. The HR (beats⋅min^−1^) was taken from the Polar app for iPhone (which was connected via Bluetooth), and perceived exertion was monitored as per the rating of Borg (6–20 (Borg et al., 1985);). Blood lactate (BLa) was assessed via a capillary sample from a fingertip on the non‐dominant hand and then analyzed using the Biosen C‐Line Glucose and Lactate analyzer (EKF Diagnostics, Cardiff, UK).

### 2.3 Treatments, randomization and blinding

The products (CBD/placebo[PLA]) used in this study (Naturecan Ltd. Stockport, UK) were both 100% vegan certified and third‐party laboratory tested for cannabinoids, residual solvents, pesticides, heavy metals and microbials (Spring Creek Labs, Utah, USA). Participants in the experimental group received a 10 mL 40% concentration broad‐spectrum OTS oral formulation of CBD (395.13 mg/g), CBG (8.83 mg/g) and other cannabinoids in trace concentrations, including ∆^9^‐THC (<0.0025% [with 0.00025% the limit of detection]) in medium‐chain triglyceride oil. The placebo group was provided with 10 mL visually identical MCT‐only oil. Participants were familiarized with supplement ingestion, which comprised self‐administering 9 drops (0.2 g/203.4 mg) of their treatment sublingually, waiting for 30 s and then swallowing. Participants were instructed to consume their respective supplement at the same time of day (∼8a.m.) +/− 1 h daily, for 3 weeks and instructed to store their respective supplements in a cool, dry place out of the reach of children. In addition, participants were instructed to consume their supplement without food, to avoid any nutrition‐related variability in CBD metabolism as high‐fat meals have been reported to increase absorption rates in humans (Perucca & Bialer, 2020). Participants were instructed to inform the lead researcher if they failed to consume a dose and that they must not “double dose” the next day, instead, informing the lead researcher. The final dose of participants' respective treatments was consumed before arrival at the laboratory on the final day of testing.

### 2.4 Nutrition

As per recommended guidelines for carbohydrate (CHO) intake on the day before high‐intensity exercise (Mata et al., 2019), participants were required to consume ∼6 g^.^kg^−1^ body mass CHO on the day before both the pre‐ and post‐intervention performance trials. Participants were also encouraged to replicate their nutritional intake from their pre‐supplementation visit in their final visit. To verify participants consumed the desired CHO, the “snap‐n‐send” method as described to be valid and reliable in recreational athletes (Costello et al., 2017). All data were analyzed by a registered sports nutritionist (SENr) using Nutritics software for Macintosh (Nutritics, Swords, Ireland). In the morning of each performance trial, participants were required to have abstained from caffeine in the 12 h before their visit with the “snap‐n‐send” method as previously described implemented for the participants' pre‐exercise breakfast. Finally, participants were instructed to avoid alcohol consumption and any strenuous exercise in the 24 h before each visit to the laboratory.

### 2.5 Blinding

Following familiarization, participants were block‐randomised into two groups, experimental (CBD) or control (PLA). Specifically, participants were matched for peak power (W) output achieved in their first familiarization visit. Thereafter, the pair was then assigned to either the CBD or PLA condition; randomization was completed using a commercially available random number generator by a staff member who was independent of the study. Upon completion of the study, participants were asked which group (CBD/PLA) they believed they were in. Twelve of the 22 (54%) participants were correct in their guess.

### 2.6 Statistical analysis

Statistical analysis was conducted in The Statistical Package for Social Sciences (SPSS; Version 27, IBM, Chicago, IL, USA) with collation and creation of figures using GraphPad Prism^TM^ for Macintosh (Version 9.3.1). All data were analyzed using two‐way ANOVA with η^2^p calculated to express the magnitude of effects. Significance was assumed if *α* reached ≤0.05. All data are presented as mean ± standard deviation.

## 3 RESULTS

Two participants were removed from this study. The first was removed due to illness unrelated to the research intervention on the final day of testing and the second because of self‐reported non‐compliance (failure to adhere to the supplementation protocol) with the study procedures. Both participants were in the experimental group (CBD), as such analysis was completed on CBD (*n* = 10) and PLA (*n* = 12). The final characteristics of the participants included in all analysis were male = 11; female = 11; age: 26.18 ± 6.2 years; stature: 170.25 ± 10.27 cm and body mass: 69.32 ± 13.56 kg.

**FIGURE 2:**
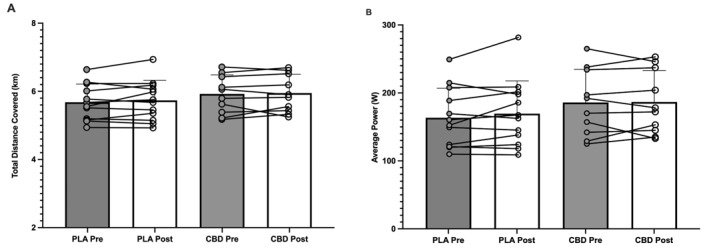
Average and individual (A) total distance covered (km) and (B) average 10‐min power (W) pre–post supplementation in the placebo and cannabidiol groups, respectively.

There was no significant effect of time (*p* = 0.341; partial Eta^2^ = 0.046) or group (*p* = 0.701; η^2^ *p* = 0.008) in total distance (km) covered pre–post supplementation in either the PLA (5.68 ± 0.54 vs. 5.74 ± 0.59 km) or CBD (5.93 ± 0.56 vs. 5.95 ± 0.52 km) groups (Figure 2A). In addition, there was no significant effect of time (*p* = 0.313; η^2^ *p* = 0.051) or group (*p* = 0.423; η^2^ *p* = 0.032) in average power (W) covered in the 10 min of the performance trial pre–post supplementation in either the PLA (163.5 ± 43.37 vs. 169.5 ± 48.36 W) or CBD (185.9 ± 48.76 vs. 186.6 ± 46.5 W) groups (Figure 2B).

**FIGURE 3:**
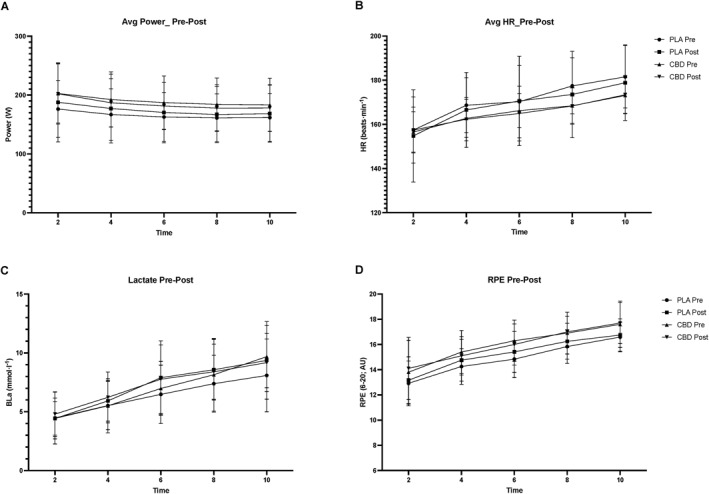
Pre–post supplementation changes in mean power (W) for PLA (A) and CBD (B). Pre–post supplementation changes in mean heart rate (b^.^min^−1^) for PLA (C) and CBD (D). Pre–post supplementation changes in blood lactate (mmol^.^L^−1^) for PLA (E) and CBD (F). Pre–post supplementation changes in RPE (AU; Borg 6–20) for PLA (G) and CBD (H). CBD, cannabidiol; PLA, placebo.

Average power (W) was not significantly changed pre–post supplementation (*p* = 0.77; η^2^ *p* = 0.004) with no time*group effect pre–post supplementation (*p* = 0.32; η^2^ *p* = 0.049). However, as expected, there was a significant effect of time point (*p* < 0.001; η^2^ *p* = 0.72) with no difference between groups at each time point (*p* = 0.494; η^2^ *p* = 0.17; Figure 3A). There was no significant effect of time for average HR (beats⋅min^−1^) pre–post supplementation (*p* = 0.528; η^2^ *p* = 0.02) and time*group pre–post supplementation (*p* = 0.571; η^2^ *p* = 0.016). However, there was a significant effect of time (*p* < 0.001; η^2^ *p* = 0.9), but this was not different between groups (*p* = 0.349; η^2^ *p* = 0.22; Figure 3B). There were no observed changes in BLa (mmol⋅l^−1^) significant effect of time for pre–post supplementation (*p* = 0.151; η^2^ *p* = 0.1) or time*group pre–post supplementation (*p* = 0.472; η^2^ *p* = 0.026). Again, as expected, there was a significant effect of time point (*p* < 0.001; η^2^ *p* = 0.92), but this was not different between groups (*p* = 0.972; η^2^p; Figure 3C). There was no significant effect of time for perceived exertion (RPE; 6–20) pre–post supplementation (*p* = 0.23; η^2^ *p* = 0.071) with no time*group pre–post supplementation effect present either (*p* = 0.19; η^2^ *p* = 0.086). Once more, there was a significant effect of time point (*p* < 0.001; η^2^ *p* = 0.82), but this was not different between groups (*p* = 0.823; η^2^ *p* = 0.081; Figure 3D).

## 4 DISCUSSION

The aim of the present study was to assess if 3 weeks of daily CBD supplementation could improve performance during a 10‐min cycling performance trial. Despite the growing popularity of CBD products, and contrary to our working hypothesis, the primary finding of this study was that chronic supplementation (150 mg⋅day^−1^) of a broad‐spectrum CBD product did not reduce perceived exertion or improve cycling performance in a 10‐min performance trial when compared to placebo.

This study was formulated from the suggestion that CBD may provide an analgesic effect in humans (De Vita et al., 2021; Urits et al., 2020). The mechanistic basis for the proposed analgesic effect of CBD is that it is a non‐competitive antagonist of both cannabinoid receptors, type 1 (CB_1_) and type 2 (CB_2_). While CB_2_ receptors are predominantly found in the periphery and cells of immune origin (Wiley & Martin, 2002; Zou & Kumar, 2018), it is important to remember that CB_1_ are abundant within the central nervous system (CNS). Indeed, CB_1_ receptors have been cited to be particularly abundant in the midbrain and spinal cord, both of which are, in part, responsible for pain perception (Manzanares et al., 2006). A further potential mechanism of analgesia is that CBD has been proposed to play a significant role in G protein‐coupled receptor 3 (GPR3) activity which is expressed within the CNS and may play a role in both pain perception and emotional regulation (Laun et al., 2019). Moreover, improved subjective exercise enjoyment has been linked to a potentially analgesic effect (Sahinovic et al., 2022). This enhanced enjoyment of exercise could perhaps be attributed to CBD's interaction with 5‐HT_1A_ receptors, which have been shown to play a role in cognition and specifically mood regulation (Albert, 2012; Sahinovic et al., 2022). Despite these potential mechanisms of pain reduction, in this exercise modality, we report no improvements in either exercise performance or ratings of perceived effort. This is unlike other “pain killing” compounds, such as tramadol (a medication prohibited by WADA from 2024), which has somewhat conflicting results on exercise, with some reports of no effect (Zandonai et al., 2021) and others of improved performance (Holgado et al., 2018; Mauger et al., 2023). Therefore, there is seemingly some contention surrounding an appropriate dose and route of administration of CBD. For example, it has been reported that CBD may provide analgesia post‐surgery with a dose as little as 25 mg (Hurley et al., 2023). Conversely, a single dose of 800 mg had no reported effect on pain (Schneider et al., 2022). In exercise models specifically, a dose of 60 mg was reported to be beneficial in recovery from muscle damage (Isenmann et al., 2020), while 150 mg exhibited no effect on non‐invasive markers of muscle damage (Cochrane‐Snyman et al., 2021). Taken together, the dose used in the present study adds further rigor to the suggestion that larger doses of CBD may be required to elicit its potential analgesic effects.

Given the broad‐spectrum products consumed in the present study did not enhance performance in this exercise modality, our observations should encourage further work in this field. Indeed, while this was a small study recruiting a modest sample size of recreational athletes, we believe this work is the first of its kind to bring into question the anti‐doping regulations of cannabinoids. It is perhaps advisable that now larger studies investigating various exercise modalities are required to encourage the consideration of anti‐doping reform. As discussed previously, all cannabinoids (except CBD) are classified as prohibited substances. The CBD used in this study was “broad‐spectrum”, containing compounds which would in principle result in an AAF by WADA. Indeed, the certificate of analysis provided by the manufacturer reported the presence of other cannabinoids, including cannabigerol (CBG), cannabichromeme (CBC) and cannabigerolic (CBGA). Whilst it is unclear if anti‐doping laboratories actively test for all cannabinoids (rather than just the intoxicating cannabinoid ∆^9^‐THC), it is crucial to remember that only one of the 11 ADRVs involves an AAF. These considerations are vital as some of the therapeutic benefits associated with CBD have been suggested to be related to the “entourage effect” (Russo, 2019). Indeed, while isolating CBD is at present the only “safe” way for an athlete to take CBD from an anti‐doping perspective, this may not be the most effective method to achieve the many benefits outlined. Future research must now fully explore this entourage effect in athletic situations as well as provide more evidence for WADA to make an informed decision on the prohibited status of other cannabinoids, investigating their relative safety, effect on performance and if their use retracts from the spirit of sport.

## 5 LIMITATIONS

While important for the growing body of literature in CBD research, this study is not without its limitations. The sample size used, although in line with other similar studies (e.g., Sahinovic et al., 2022), was limited by logistical and practical considerations, such as financial cost and time constraints; both aspects generally accepted to impact on subject recruitment (Lakens, 2022). In addition, given that the present study did not have a large enough sample size to complete a sub‐group sex analysis, it is not clear whether CBD supplementation affects the physiological response to exercise in the same way for both sexes, and therefore, it is difficult to postulate whether the results could have been affected by the inclusion of both sexes. Future studies should examine sex differences in CBD supplementation responses in such parameters. Nonetheless, the study provides useful information to further research into the area of broad‐spectrum CBD supplementation in exercising individuals.

## 6 PRACTICAL APPLICATIONS

This study provides athletes and associated personnel with novel data on the potential utility of broad‐spectrum CBD supplements in reducing perceived exertion during aerobic exercise. Our findings demonstrate that the commercially available CBD supplement used in our study does not enhance performance during a 10‐min cycle‐ergometer performance trial. Given that there are anti‐doping risks associated with CBD supplementation, coupled with the lack of any clear benefit to performance, it is advisable for athletes to maintain abstinence from CBD supplementation.

## 7 CONCLUSION

Our data suggests that a broad‐spectrum CBD supplement (containing <0.0025% ∆^9^‐THC but including traces of other cannabinoids including CBG, CBC and CBGA) did not enhance performance during a 10‐min cycle ergometer performance trial. Given the concerning data outlining the disparity between advertised and actual cannabinoid content claims of OTS CBD products, coupled with only one cannabinoid being considered as a threshold substance, athletes should maintain abstinence of OTS CBD supplements. Research should now be performed to investigate if a range of other cannabinoids has the potential to influence performance in a variety of exercise modalities.

## CONFLICT OF INTEREST STATEMENT

No conflicts of interest to disclose.

## Supporting information

Figure S1
