# Supplementary figures and images for: Does a broad‐spectrum cannabidiol supplement improve performance in a 10‐min cycle ergometer performance‐test?
Source: Eur J Sport Sci. 2024 May 3;24(7):870–7. doi: 10.1002/ejsc.12116 (PMC11236031; doi:10.1002/ejsc.12116)

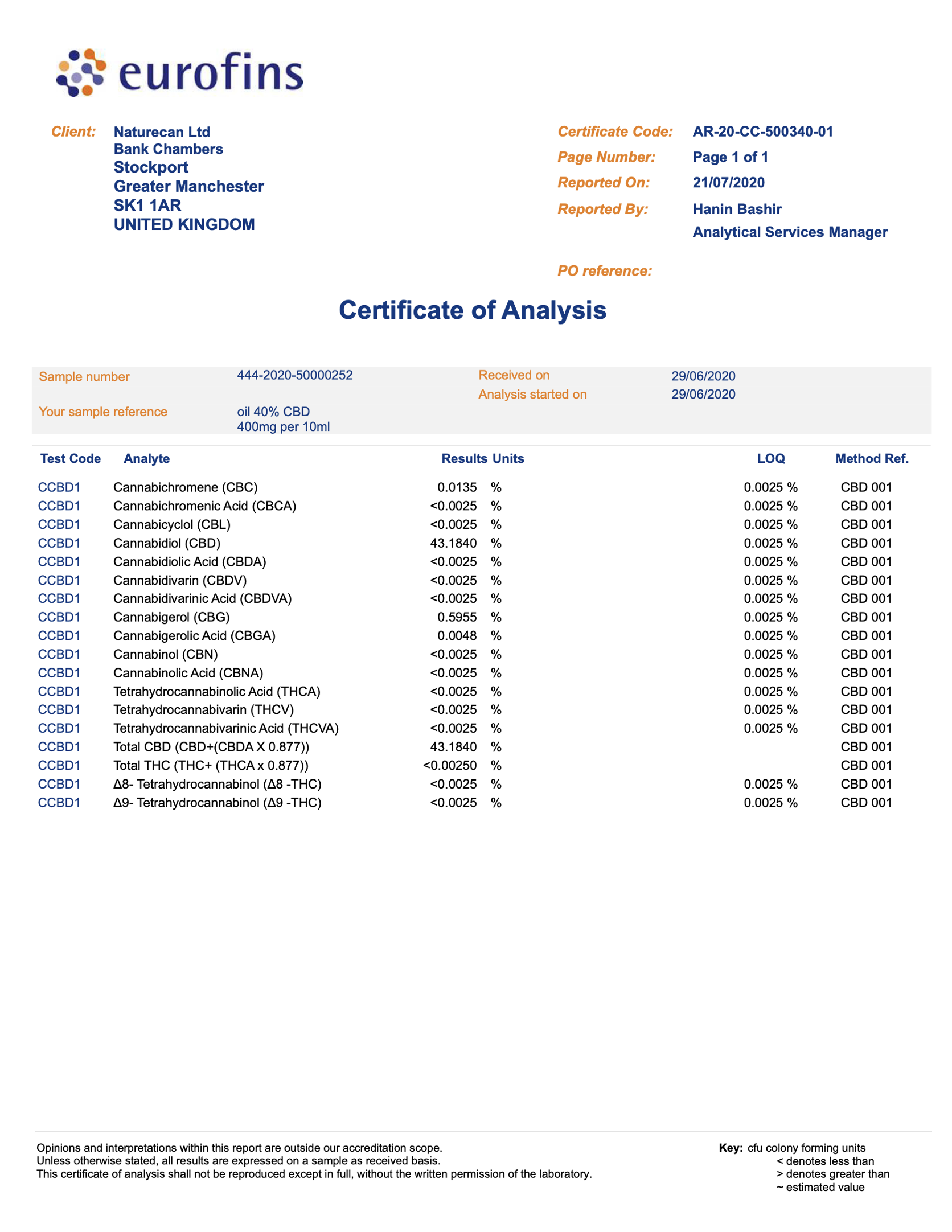

Supplement: Supplementary file 1 — Figure S1 [file EJSC-24-870-s001.png]
